# Plant kinetochore complex: composition, function, and regulation

**DOI:** 10.3389/fpls.2024.1467236

**Published:** 2024-10-10

**Authors:** Yuqian Xie, Mingliang Wang, Beixin Mo, Chao Liang

**Affiliations:** ^1^ Guangdong Provincial Key Laboratory for Plant Epigenetics, College of Life Sciences and Oceanography, Shenzhen University, Shenzhen, China; ^2^ Synthetic Biology Research Center, Shenzhen University, Shenzhen, China

**Keywords:** centromere, kinetochore complex, CENH3, CENP-C, cenDNAs, cenRNAs, plant artificial chromosomes

## Abstract

The kinetochore complex, an important protein assembly situated on the centromere, plays a pivotal role in chromosome segregation during cell division. Like in animals and fungi, the plant kinetochore complex is important for maintaining chromosome stability, regulating microtubule attachment, executing error correction mechanisms, and participating in signaling pathways to ensure accurate chromosome segregation. This review summarizes the composition, function, and regulation of the plant kinetochore complex, emphasizing the interactions of kinetochore proteins with centromeric DNAs (cenDNAs) and RNAs (cenRNAs). Additionally, the applications of the centromeric histone H3 variant (the core kinetochore protein CENH3, first identified as CENP-A in mammals) in the generation of ploidy-variable plants and synthesis of plant artificial chromosomes (PACs) are discussed. The review serves as a comprehensive roadmap for researchers delving into plant kinetochore exploration, highlighting the potential of kinetochore proteins in driving technological innovations in synthetic genomics and plant biotechnology.

## 1 Introduction

**Figure 1:**
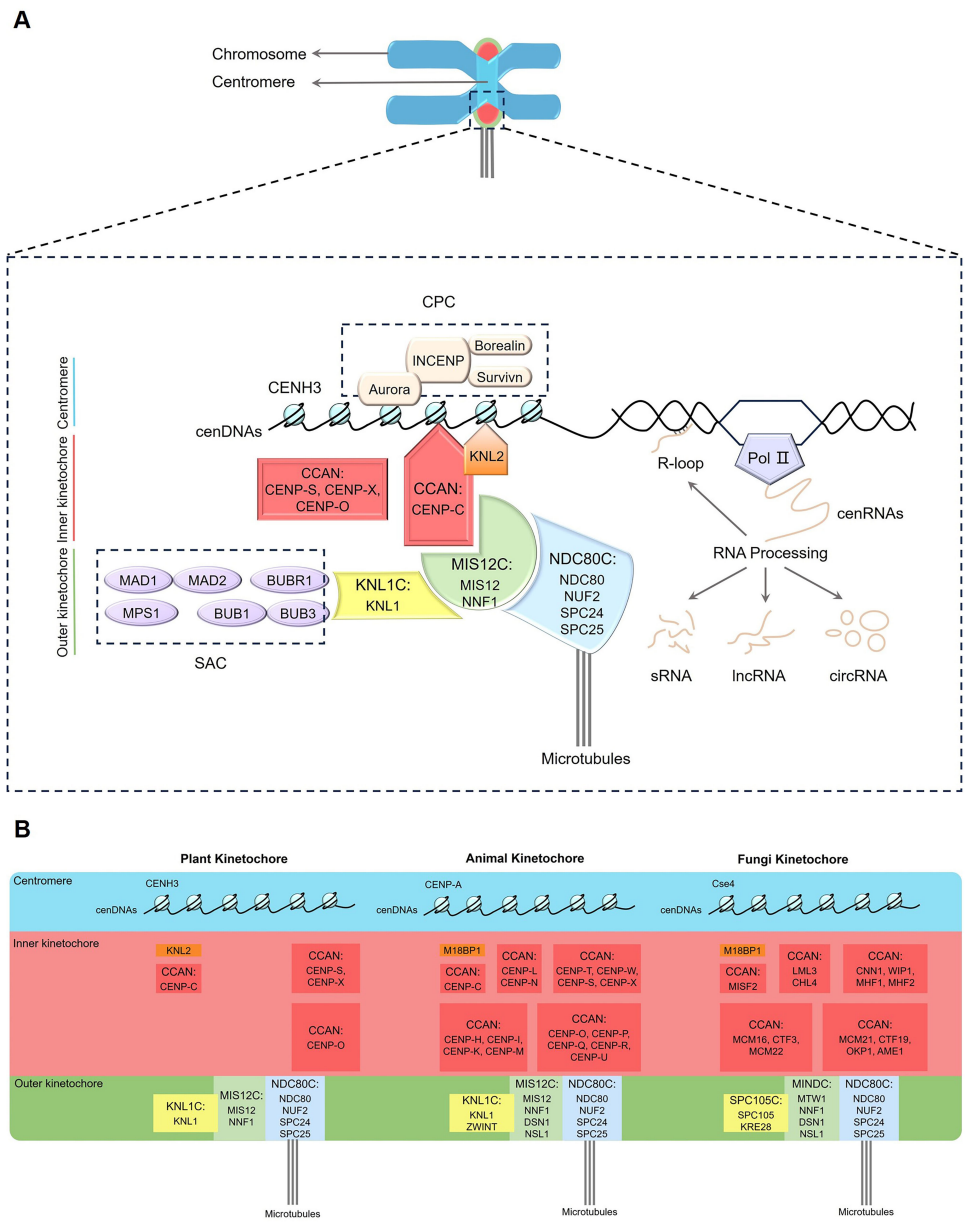
Schematic of the plant kinetochore complex. **(A)** Plant kinetochore region. The red and orange shading represent the inner kinetochore components, while the yellow, green and blue shading refer to the outer kinetochore components. **(B)** Comparison of kinetochore composition in plants, animals and fungi. Pol II, RNA Polymerase II; CCAN, constitutive centromere-association network; CENH3, centromeric histone H3 variant; CENP-C, centromere protein C; KNL2, kinetochore null2; SAC, spindle assembly checkpoint; CPC, chromosome passenger complex; KNL1C, kinetochore scaffold 1 complex; MIS12C, minichromosome instability 12 complex; NDC80C, nuclear division cycle 80 complex; cenDNAs, centromeric DNAs; cenRNAs, centromeric RNAs; R-loop, a special chromosome structure that contains one strand of single-stranded DNA and another strand comprising a DNA, RNA hybrid; sRNA, small RNA; lncRNA, long non-coding RNA; circRNA, circular RNA.

The centromere, a specific region located in the primary constriction of a chromosome, plays a crucial role in cell division. It participates in connecting sister chromatids and facilitating chromosome segregation during mitosis and meiosis (Sundararajan and Straight, 2022; Evatt et al., 2024). According to the size and distribution, centromeres can be divided into four classes, point centromere, holocentromere, monocentromere and metapolycentromere (Ekwall, 2007; Oliveira and Torres, 2018; Plačková et al., 2021; Kuo et al., 2023). This review focuses on plant monocentromeres, while other types of centromeres are beyond its scope. The kinetochore is a giant protein complex assembled on the centromere of eukaryotes, with more than 100 structural and regulatory proteins involved in its assembly (McAinsh and Marston, 2022; Ariyoshi and Fukagawa, 2023). Each chromosome contains two kinetochores on either side of the centromere during metaphase (**Figure 1A**). Electron micrograph shows that the kinetochore is a disc-shaped structure including inner and outer layers (Dawe et al., 2005). The inner part is intertwined with the centromere, while the outer part is mainly used for spindle microtubule attachment (Dawe et al., 2005; Cheeseman, 2014; Monda and Cheeseman, 2018). The kinetochore complex is structurally intricate, with various protein components having extensive interactions. In addition to their mutual interactions, the kinetochore proteins frequently bind to centromeric DNAs (cenDNAs) and RNAs (cenRNAs) (Du et al., 2010; Sandmann et al., 2017; Wlodzimierz et al., 2023; Fernandes et al., 2024). During cell division, the kinetochore complex serves as an interface between chromosomes and spindle microtubules (MTs), facilitating the precise movement and segregation of chromosomes. This plays a crucial role in signal transduction, monitoring the correct attachment of chromosomes to spindle MTs as well as regulating the progression of the cell cycle by shaping the structure and morphology of chromosomes, thereby ensuring their proper alignment and segregation (Cheeseman, 2014; Monda and Cheeseman, 2018; Cairo and Lacefield, 2020). Similar to its counterparts in animals and fungi, the kinetochore complex in plant cells performs vital functions such as maintaining the structural stability of chromosomes, regulating microtubule attachment and dynamics, participating in error correction mechanisms, as well as taking part in the regulation of signaling pathways and checkpoint recognition (Kixmoeller et al., 2020; Zhou et al., 2023). These functions collectively ensure the accuracy of cytokinesis and the stable transmission of genetic information (Cheeseman, 2014; Zhou et al., 2023).

In this review, the composition, function, and regulation of the plant kinetochore complex are summarized, with details on the interactions of the kinetochore proteins with cenDNAs and cenRNAs. Additionally, the applications of the core kinetochore protein CENH3, the centromeric histone H3 variant, in the generation of ploidy-variable plants and the synthesis of plant artificial chromosomes (PACs), mainly in *Arabidopsis thaliana*, are described. This comprehensive review serves as a roadmap for researchers embarking on the journey of plant kinetochore exploration by systematically providing current knowledge of the kinetochore complex, identifying research gaps, and proposing future directions. The review aims to inspire innovative approaches that can advance the field of plant kinetochore research, thereby guiding and inspiring future inquiries in this crucial research area.

## 2 Composition and interaction network of the kinetochore complex

The kinetochore complex consists of a variety of proteins, including core kinetochore proteins and associated proteins that interact and coordinate in orchestrating chromosome movement and segregation processes (McAinsh and Marston, 2022). Based on the position and functions, the core subunits of kinetochore are classified into two main parts, namely the inner constitutive centromere-association network (CCAN) and the outer KNL1 complex (kinetochore scaffold 1 complex, KNL1C), MIS12 complex (minichromosome instability 12 complex, MIS12C), and NDC80 complex (nuclear division cycle 80 complex, NDC80C) network (KMN) (**Figure 1**, **Table 1**) (McKinley and Cheeseman, 2016; Pesenti et al., 2016; Comai et al., 2017; Yatskevich et al., 2022; Zhou et al., 2023; Yatskevich et al., 2024). The inner CCAN is located within the centromeres throughout the cell cycle, while the outer KMN is recruited to centromeres specifically during the M phase (mitotic phase), when nuclear and cytoplasmic divisions occur, leading to the production of two daughter cells (Hara and Fukagawa, 2017). The inner CCAN mainly binds to CENH3 nucleosomes, while the outer KMN connects directly to MTs, mediating the interaction between MTs and the inner CCAN (Lermontova et al., 2013; Yatskevich et al., 2023; Zhou et al., 2023; Deng et al., 2024a, 2024b). Additionally, the kinetochore proteins also oversee the spindle assembly checkpoint (SAC), assisting in the accurate alignment of chromosome and the successful completion of mitosis (Lara-Gonzalez et al., 2021; Deng et al., 2024a, 2024b). The loading and functioning of SAC on kinetochores also depend on the chromosome passenger complex (CPC) (Komaki et al., 2020; Zhou et al., 2023). In addition to interacting with each other, kinetochore proteins often bind to cenDNAs and cenRNAs (Du et al., 2010; Sandmann et al., 2017; Wlodzimierz et al., 2023; Fernandes et al., 2024).

**Table 1:** List of plant kinetochore proteins^a^.

Name	Homologs	References
CENH3	AtCENH3, ZmCENH3, PpCENH3	(Lermontova et al., 2006, 2011; Maruthachalam et al., 2011; Kozgunova et al., 2019; Feng et al., 2020; Capitao et al., 2021)
CENP-C	AtCENP-C, ZmCENP-C, PpCENP-C	(Ogura et al., 2004; Zheng et al., 2017; Kozgunova et al., 2019; Zhou et al., 2023)
CENP-S	AtCENP-S (MHF1), PpCENP-S	(Dangel et al., 2014; Kozgunova et al., 2019)
CENP-X	AtCENP-X (MHF2), PpCENP-X	(Dangel et al., 2014; Kozgunova et al., 2019)
CENP-O	PpCENP-O	(Kozgunova et al., 2019)
KNL2	αKNL2, βKNL2, γKNL2, δKNL2	(Lermontova et al., 2013; Boudichevskaia et al., 2019; Zuo et al., 2022)
KNL1	AtKNL1, ZmKNL1, PpKNL1	(Kozgunova et al., 2019; Su et al., 2021; Deng et al., 2024a)
MIS12	AtMIS12, ZmMIS12	(Sato et al., 2005; Li and Dawe, 2009)
NNF1	AtNNF1	(Allipra et al., 2022)
NDC80	ZmNDC80	(Du and Dawe, 2007)
NUF2	AtNUF2	(Li et al., 2021)
SPC24	AtMUN	(Shin et al., 2018)
SPC25	AtSPC25	(Shin et al., 2018; Li et al., 2021)
MPS1	AtMPS1, OsPRD2	(Jiang et al., 2009; Wang et al., 2023a)
BUB1 (BMF1)	ZmBUB1, OsBRK1	(Wang et al., 2012; Su et al., 2017)
BUB3	AtBUB3.1, AtBUB3.2, AtBUB3.3, ZmBUB3	(Su et al., 2017; Zhang et al., 2018, 2021; Lermontova et al., 2008)
BUBR1 (MAD3)	AtMAD3.1 (BMF2), AtMAD3.2 (BMF2)	(Caillaud et al., 2009; Komaki and Schnittger, 2016)
MAD1	AtMAD1	(Bao et al., 2014)
MAD2	AtMAD2, ZmMAD2, TaMAD2	(Yu et al., 1999; Caillaud et al., 2009; Bao et al., 2014)
Aurora	AtAURORA1, AtAURORA2, AtAURORA3	(Demidov et al., 2005; Weimer et al., 2016; Komaki et al., 2020; Deng et al., 2024c)
INCENP	AtWYR	(Kirioukhova et al., 2011; Komaki et al., 2020)
Borealin	AtBORR	(Kirioukhova et al., 2011; Komaki et al., 2020)
Survivin	AtBORI2	(Komaki et al., 2020, 2022)

a At, *Arabidopsis thaliana*; Os, *Oryza sativa*; Zm, *Zea mays*; Pp, *Physcomitrella patens*; Ta, *Triticum aestivum*.

### 2.1 CENH3 protein and inner kinetochore proteins

#### 2.1.1 CENH3 protein

**Figure 2:**
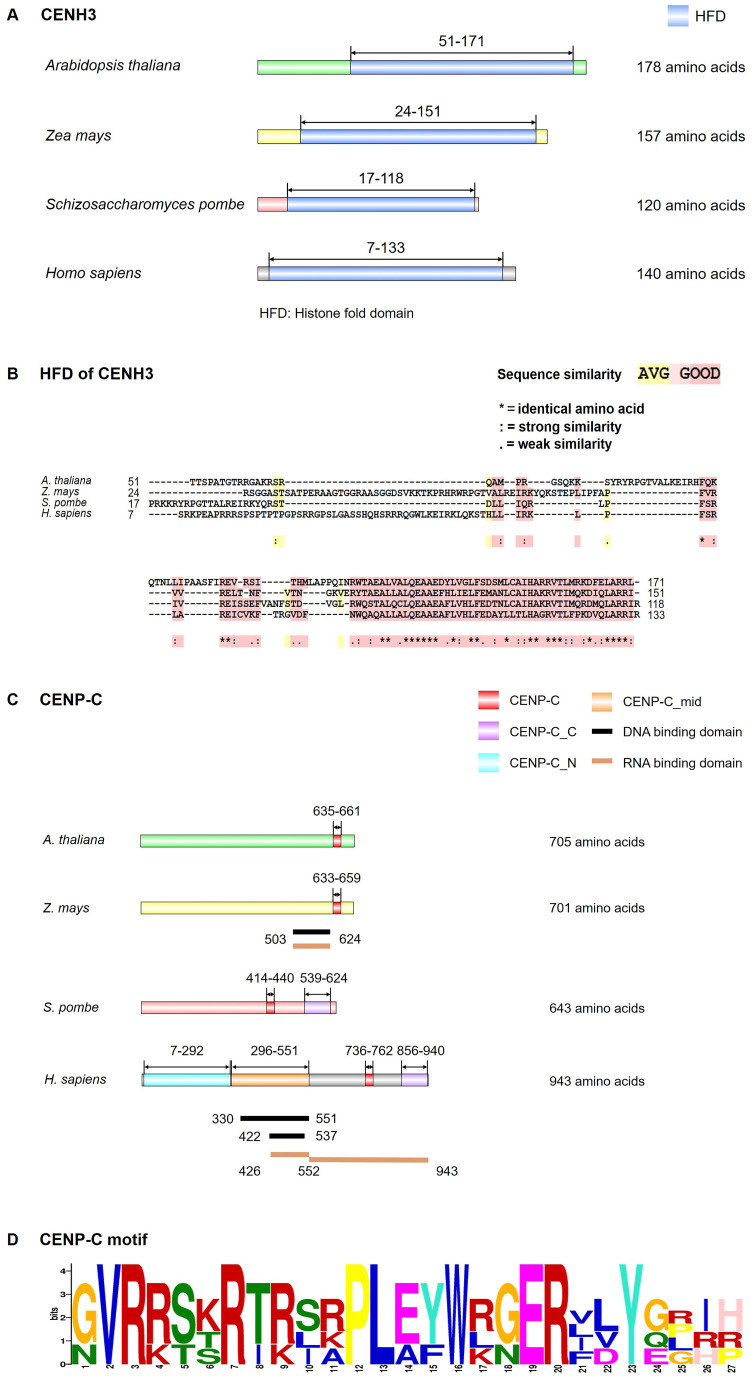
Structural model of CENH3 and CENP-C. **(A)** Conserved motif of CENH3 in *Arabidopsis thaliana*, *Zea mays*, *Schizosaccharomyces pombe* and *Homo sapiens* by InterPro (https://www.ebi.ac.uk/interpro/) and IBS software. **(B)** Multiple protein sequence alignment of CENH3 histone fold domain (HFD) in *A. thaliana*, *Z. mays*, *S. pombe* and *H. sapiens* was generated by the T-COFFEE program. The color code indicates the similarity between the protein sequences as indicated. **(C)** Conserved motif, DNA binding domain and RNA binding domain of CENP-C in *A. thaliana*, *Z. mays*, *S. pombe* and *H. sapiens* by InterPro website and IBS software. **(D)** Logos representation of an alignment of the CENP-C motif in *A. thaliana*, *Z. mays*, *S. pombe* and *H. sapiens* through MEME (https://meme-suite.org/meme/tools/meme). Data used were from the references (Yang et al., 1996; Sugimoto et al., 1997; Talbert et al., 2004; Wong et al., 2007; Du et al., 2010; Wang et al., 2023b).

CENH3 protein is important for chromosome segregation, with its proper deposition being a prerequisite for the correct assembly of kinetochore components. The name of CENH3 is various in different organisms, namely CENP-A in animals and fission yeast, Cse4 in budding yeast, and CENH3 in plants and many protists (Talbert and Henikoff, 2018). Interspersed with canonical H3, CENH3 forms nucleosomes with cenDNAs at the centromere (Kixmoeller et al., 2020). CENH3 is widely present in plants, with its function being evolutionarily conserved across various species. Like conventional H3, CENH3 has a N-terminal tail domain (protruding from the nucleosome, serving as a target for post-translational modifications) and a conserved C-terminal histone-fold domain (HFD) (**Figures 2A, B**). The N-terminal of CENH3 exhibits high variability in the length and sequence of amino acids (Raipuria et al., 2023), while the C-terminal HFD is conserved in most eukaryotes. Though mitotic chromosome segregation is supported by either the CENH3 N-terminal or the histone H3 N-terminal, normal functioning of CENH3 requires its N-terminal (Ravi et al., 2010). In *Arabidopsis*, the C-terminal of CENH3 is sufficient for the loading of CENH3 during mitosis (Lermontova et al., 2006), but meiotic loading requires both the C and N-terminals (Lermontova et al., 2011; Maruthachalam et al., 2011). The C-terminal HFD of *Arabidopsis* CENH3 can be loaded onto the centromeres in the absence of its N-terminal during mitosis (Lermontova et al., 2006). However, N-terminal truncated CENH3 cannot be loaded onto the centromeres of the meiotic nucleus, leading to chromosome lag and micronucleus formation, thereby reducing plant fertility (Lermontova et al., 2011). In contrast, heterologous CENH3 from some grass species may be able to localize at centromeres in the presence of the native *Arabidopsis* CENH3, but are not functionally competent in the absence of native CENH3, indicating that the localization of CENH3 is not necessarily associated with its centromere function (Ravi et al., 2010). The C-terminal tail of maize (*Zea mays*) CENH3 could bind to histone H4, resulting in the formation of stable nucleosomes (Feng et al., 2020). Using RNA interference (RNAi) to knockdown *CENH3* in *Arabidopsis* resulted in abnormal mitosis, ultimately leading to dwarf plants (Lermontova et al., 2011). Capitao et al. showed that *Arabidopsis* plants, partially deficient in the RNA decay factor - suppressor with morphogenetic effects on genitalia 7 (SMG7), failed to exit meiosis, causing diminished fertility. *CENH3* mutation in SMG7-deficient plants promoted the exit of meiosis and the restoration of fertility (Capitao et al., 2021).

#### 2.1.2 Inner kinetochore proteins

In most eukaryotes, CENH3 physically interacts with the inner CCAN (Cortes-Silva et al., 2020; Fellmeth and McKim, 2020; Carty et al., 2021). Based on the functions, spatial location, and recruitment relationship, the 16 subunits of vertebrate inner CCAN are divided into several subcomplexes, namely CENP-C (centromere protein C), CENP-H/I/K/M, CENP-L/N, CENP-T/W/S/X, and CENP-O/P/Q/R/U (Pesenti et al., 2016). Among these, CENP-C serves as a basic kinetochore protein that binds to CENH3 nucleosomes, DNAs, and RNAs (Kerstin et al., 2015; Walstein et al., 2021; Ariyoshi and Fukagawa, 2023). It occupies a pivotal position in the recruitment of other kinetochore proteins, with a conserved CENP-C motif and ubiquitous interactions with other kinetochore proteins (**Figures 2C, D**). In humans (*Homo sapiens*), the N-terminal of CENP-C interacts with the MIS12C of the outer KMN, bridging the inner CCAN and the outer KNM (Kerstin et al., 2015; Ariyoshi and Fukagawa, 2023; Hara et al., 2023). The middle part of CENP-C interacts with the other components of CCAN (Kerstin et al., 2015; Ariyoshi and Fukagawa, 2023; Hara et al., 2023), while the C-terminal of CENP-C harbors a cupin domain that facilitates protein dimerization, CENP-C’s centromere localization, and interaction with CENP-A. CENP-C is the only kinetochore component in plants homologous to vertebrate CCAN (Zhou et al., 2023), which was found in *Arabidopsis* (Ogura et al., 2004), maize (Zheng et al., 2017), and moss (*Physcomitrella patens*) (Kozgunova et al., 2019). In addition, the moss possesses CENP-S, CENP-X, and CENP-O, but these proteins are not localized to kinetochores (Kozgunova et al., 2019). Despite the absence of kinetochore enrichment in moss CENP-X, RNAi-mediated knockdown of CENP-X resulted in chromosome missegregation and cytokinesis failure, which could be rescued by ectopic expression of CENP-X (Kozgunova et al., 2019). This proves that CENP-X is essential for chromosome segregation and cell division (Kozgunova et al., 2019). Like humans, plant CENP-S (MHF1) and CENP-X (MHF2) interact with Fanconi anaemia complementation group M (FANCM) by forming a DNA remodeling complex, thereby participating in the damage-dependent DNA binding of FANCM (Dangel et al., 2014; Singh et al., 2023). Besides, in chicken DT40 cells, they also play important roles in the establishment of kinetochore functions to ensure proper chromosome segregation (Amano et al., 2009; Nishino et al., 2012). Though plant CENP-S and CENP-X are related to DNA repair and homologous recombination (Dangel et al., 2014; Singh et al., 2023), their specific roles in kinetochore assembly remain elusive.

Kinetochore null2 (KNL2, also known as Mis18 binding protein 1, M18BP1 in animals) is another important inner kinetochore protein (Subramanian et al., 2014; Hori et al., 2017; Sandmann et al., 2017; Zuo et al., 2022; London et al., 2023). Currently, all known KNL2 proteins harbor a conserved domain called Swi3-Ada2-NCoR-TFIIIB-associated (SANTA). However, in *Xenopus laevis*, SANTA was found unessential for the interaction between M18BP1 and CENP-A nucleosomes, whereas M18BP1 was observed to bind to CENP-C through SANTA (French and Straight, 2019). Moreover, *Arabidopsis* KNL2 was found to target centromeres and interact with DNA independently of SANTA (Lermontova et al., 2013; Sandmann et al., 2017). Thus, the precise role of SANTA in KNL2 remains enigmatic. Further, the CENPC-like (CENPC-k) conserved motif is also present in the C-terminal region of eukaryotic KNL2 homologs, including those of *X. laevis* (French et al., 2017), chicken (Hori et al., 2017), and *Arabidopsis* (Sandmann et al., 2017; Zuo et al., 2022). In plants, the *KNL2* gene is divided into two branches, namely *αKNL2* and *βKNL2* in eudicots, and *γKNL2* and *δKNL2* in grasses. Only *αKNL2* and *γKNL2* were found to have CENPC-k (Zuo et al., 2022). *Arabidopsis* has both *αKNL2* and *βKNL2*, while maize has only *δKNL2* (Zuo et al., 2022). Mutations in *Arabidopsis KNL2* result in mitotic and meiotic defects and reduced DNA methylation, resulting in slow development and impaired reproductive capability (Boudichevskaia et al., 2019).

#### 2.1.3 Interactions among CENH3, CENP-C, and KNL2 in plants

Plant centromere recognition and functions are epigenetically defined by CENH3, serving as the basis of kinetochore formation (Naish and Henderson, 2024). CENH3 combines with double-stranded DNA to form CENH3 nucleosomes, achieved through local high concentrations of CENH3 (McKinley and Cheeseman, 2016). Chromosome segregation leads to the halving of parental histones, counterbalanced by the loading of new histones. In plants, the loading of CENH3 onto centromeres occurs during G2 and/or prophase (Lermontova et al., 2006), mainly dependent on the centromere-licensing factors, CENP-C and KNL2, and the chaperone molecule NASP/Sim3 (Le Goff et al., 2019; Stirpe and Heun, 2023). Moreover, γ-tubulin complex protein 3-interacting proteins (GIPs), GIP1 and GIP2, are required for CENH3 loading and/or maintenance (Batzenschlager et al., 2015).

The conserved CENP-C motif plays an important role in the centromere localization of CENP-C and its interaction with CENH3 in animals and fungi (Talbert et al., 2004; Nagpal et al., 2015; Ariyoshi and Fukagawa, 2023). Moreover, the CENP-C motif is the only reported conserved domain in plants to be homologous with CENP-C in animals and fungi (Talbert et al., 2004), suggesting that it may have similar functions in plant. *Arabidopsis* (α)KNL2, on the other hand, is responsible for centromere recognition and deposition of new CENH3, competing with CENP-C in this process (Lermontova et al., 2013; French et al., 2017). *Arabidopsis* (α)KNL2 could be co-localized with CENH3 at all stages of the cell cycle except metaphase and mid-anaphase, binding to CENH3 nucleosomes through its C-terminal CENPC-k conserved domain to participate in centromere recognition and CENH3 localization (Sandmann et al., 2017). This is similar to fission yeast, but different from humans where CENH3 is only present transiently in centromeres after mitotic exit (Hayashi et al., 2004; Fujita et al., 2007; Maddox et al., 2007). However, in *Arabidopsis* and *Nicotiana benthamiana*, although the centromere localization of CENP-C and (α)KNL2 is dependent on the CENPC/CENPC-k motifs, the CENPC and CENPC-k motifs alone are not sufficient (Yalagapati et al., 2024). The knockout of *Arabidopsis (α)KNL2* triggers a decrease in CENH3 transcript levels, controlling the epigenetic regulation of CENH3 assembly (Lermontova et al., 2013). However, the knockout of (α)*KNL2* reduced CENH3 assembly on centromeres but it does not abolish the centromeric localization of CENH3 (Lermontova et al., 2013). Compared to αKNL2 with CENPC-k, in eudicots, the centromere localization of βKNL2, which lacks the CENPC-k domain, may be achieved through binding to CENP-C by its SANTA domain as revealed in studies on *X. laevis* (French et al., 2017), or by its N-terminal conserved domain located upstream of the SANTA domain as revealed in studies on humans (Stellfox et al., 2016), or by a combination of these two regions. Yadala et al. proposed that the centromere localization of *Arabidopsis* βKNL2 requires its SANTA domain and the C-terminal motif-III, and depends on αKNL2 in a tissue-dependent manner (Yadala et al., 2024).

NASP/Sim3, an *Arabidopsis* ortholog of the mammalian nuclear autoantigenic sperm protein (NASP) and *Schizosaccharomyces pombe* histone chaperone Sim3, was demonstrated to bind to CENH3, affecting its abundance at centromeres (Le Goff et al., 2019). Additionally, *Arabidopsis* GIPs could form a protein complex with CENH3 and is involved in CENH3 stabilization and centromere cohesion (Batzenschlager et al., 2015). The *gip1gip2* knockdown mutant leads to a decreased CENH3 level and impaired recruitment of CENP-C at centromeres, despite a higher level of KNL2 present at both centromeric and ectopic sites (Batzenschlager et al., 2015).

### 2.2 Outer kinetochore proteins

The outer KMN physically connects the centromere and the inner CCAN to MTs, mediating the MTs-CCAN interaction for chromosome localization and segregation. The outer KMN begins to accumulate in kinetochores during prophase, remains there during interphase (Espeut et al., 2012; Deng et al., 2024a). KMN supports chromosome movement and segregation in addition to functioning as a platform for recruiting other regulatory proteins (London and Biggins, 2014; Hara and Fukagawa, 2017). The outer KMN consists of the KNL1 complex (KNL1C), MIS12 complex (MIS12C) and NDC80 complex (NDC80C) (Li and Dawe, 2009; Su et al., 2021; Neumann et al., 2023; Polley et al., 2023; Kim et al., 2024; Oliveira et al., 2024; Polley et al., 2024). Due to the similar rapid evolution of other kinetochore proteins, such as CENH3 and CENP-C (Talbert et al., 2004; Pontremoli et al., 2021), only a few KMN proteins homologous to mammals and yeast have been identified in plants.

KNL1C is comprised of KNL1 and ZWINT (ZW10 interactor), but only KNL1 has been identified and functionally characterized in plants (Su et al., 2021; Deng et al., 2024a). Two distinct functional domains - the coiled-coil domain and the RING finger, WD repeat, DEAD-like helicases domain (RWD) - connect plant KNL1 with its animal orthologs (van Hooff et al., 2017; Kozgunova et al., 2019; Su et al., 2021). Plant KNL1 is involved in the segregation of sister chromatids during mitosis (Kozgunova et al., 2019; Su et al., 2021; Deng et al., 2024a). The loss of function of KNL1 in maize led to abnormal chromosome behavior during early endosperm development, causing kernel defects (Su et al., 2021). *KNL1* mutation in *Arabidopsis* resulted in seed abortion, with the mutant plants being extremely short, forming dark/purple rosettes with deformities, and leaf trichomes with an increase in the number of branches (Deng et al., 2024a). While the homolog of human ZWINT has not yet been reported in plants, it is known to correct misattachment between kinetochores and MTs to maintain genome stability, in addition to serving as a biomarker for tumor research (Lin et al., 2021).

The members of MIS12C include MIS12, necessary for nuclear function 1 (NNF1, also known as polyamine modulated factor 1, PMF1 in humans), dosage suppressor of NNF1 (DSN1), and non-specific lethal 1 (NSL1), of which MIS12 and NNF1 were found in plants (Sato et al., 2005; Li and Dawe, 2009; Allipra et al., 2022). *Arabidopsis* MIS12 was found to interact with NNF1, localizing to the kinetochores (Allipra et al., 2022). In *Arabidopsis*, *MIS12* mutation resulted in embryo lethality and slow growth (Sato et al., 2005). Embryos and syncytial endosperms of *Arabidopsis NNF1* mutants harbored multiple nucleoli of varying sizes (Allipra et al., 2022). Further, *Arabidopsis* NNF1 was also found to affect polyamine and gibberellic acid (GA) metabolism (Allipra et al., 2022).

NDC80C is composed of NDC80, NUF2 (nuclear filament-containing protein 2), spindle pole component 24 (SPC24), and spindle pole components 25 (SPC25). All four members have been identified in plants (Du and Dawe, 2007; Shin et al., 2018; Li et al., 2021). In human, MIS12 binds to NDC80C which in turn directly interacts with MTs. NDC80 directly interacts with MIS12C, facilitating its indirect interaction with CENH3 and CENP-C (Cheeseman and Desai, 2008). Similar to animals and fungi, maize NDC80 was found to be stably maintained at centromeres during cell division (Du and Dawe, 2007). In *Arabidopsis*, NUF2, along with meristem unstructured (MUN or SPC24), and SPC25 have been found to co-localize with CENH3 (Shin et al., 2018; Li et al., 2021). Furthermore, Marimuthu et al. demonstrated that *Arabidopsis* NUF2 and CENPC colocalize with CENH3, although they did not investigate their physical interaction (Marimuthu et al., 2021). Plant NUF2 regulates spindle microtubule organization and chromosome segregation during mitosis (Li et al., 2021). Mutations in *Arabidopsis NUF2* caused severe mitotic defects in embryos, endosperm, and even seedlings, manifested as seed abortion and seedling growth cessation (Li et al., 2021). MUN affects plant development through cell division (Shin et al., 2018). *Arabidopsis MUN* mutant plants exhibited unstructured shoot apical meristem (SAM) and ectopic development of SAM (Shin et al., 2018). In addition, *Arabidopsis* SPC25 was found to co-localize with CENH3 and MTs at different stages of mitosis, suggesting that SPC25 may perform a similar function to NUF2 (Li et al., 2021).

### 2.3 Spindle assembly checkpoint proteins and chromosome passenger complex proteins

#### 2.3.1 Spindle assembly checkpoint proteins

SAC signaling is a conserved regulatory mechanism in centromeres that controls cell cycle and genome stability (Lara-Gonzalez et al., 2021; McAinsh and Kops, 2023). The core SAC proteins are composed of monopolar spindle 1 (MPS1), budding uninhibited by benomyl (BUB, including BUB1 and BUB3), BUB1-related protein 1 (BUBR1, also called MAD3), and mitotic arrest deficient (MAD, including MAD1 and MAD2) (Lara-Gonzalez et al., 2021; Zhou et al., 2022; Deng et al., 2024b). In *Arabidopsis*, MPS1 has been found to regulate the double-strand break (DSB) during meiosis and is a key factor in determining spindle bipolarity (Jiang et al., 2009). Conversely, the homolog of MPS1 in rice (*Oryza sativa*) was found to be involved only in DSB and not in spindle assembly (Wang et al., 2023a), suggesting that MPS1 may have multiple functions in different plants. Maize BUB1/BMF1 was found to mediate H2AThr133 phosphorylation within CENH3 nucleosomes (Su et al., 2017) and BUB1-related kinase BRK1 in rice ensured proper tension between homologous kinetochores during meiosis metaphase (Wang et al., 2012). MAD3.1/BMF2 and MAD3.2/BMF3 are the two MAD3/BUBR1 homologs in *Arabidopsis* (Komaki and Schnittger, 2016). Plant BUB3 contains WD40 repeats, but all BUB1/MAD3 family proteins (BMF1/2/3) lack the canonical Gle-binding site (GLEBS) domain that interacts with BUB3 (Komaki and Schnittger, 2016). *Arabidopsis* BUB3 has three homologs, BUB3.1, BUB3.2, and BUB3.3 (Lermontova et al., 2008). During cell division, *Arabidopsis* BUB3.1 and BUB3.2 were reported to regulate microtubule reorganization signal and phragmoplast development (Zhang et al., 2018, 2021). Plant BMF2 localizes to kinetochores under microtubule-destabilizing conditions to directly interact with BMF3 in kinetochores (Caillaud et al., 2009). In animals and yeast, MAD1 is localized predominantly at unattached kinetochores, recruiting MAD2 to form a MAD1–MAD2 complex (Bao et al., 2014). However, the kinetochore localization of plant MAD1 is yet to be reported. Nevertheless, plant MAD2 homologs were reported to strongly accumulate in kinetochores under microtubule-destabilizing conditions (Caillaud et al., 2009). *Arabidopsis* MAD1 was reported to regulate endopolyploidization and flowering time, indicating that MAD1 regulates cell cycle control during reproductive transition (Bao et al., 2014).

Conserved from yeast to humans, *Arabidopsis* BUB3.1, MAD2, and MAD3 were found to physically interact with each other (Caillaud et al., 2009). *Arabidopsis* KNL1 interacts with BUB3.3 and BMF3 through the eudicot-specific-domain (ESD), regulating the localization of BUB3.3 and BMF3 in kinetochores and affecting their functions in SAC signaling (Deng et al., 2024a). The absence of BUB3.3 and BMF3 from the kinetochore results in inadequate SAC signaling, halting mitotic cells from progressing to anaphase due to the lack of proper chromosomal alignment (Deng et al., 2024a). Su et al. found that a 145-amino-acid region in the middle of maize KNL1 interacted with the SAC component BMF1/2, but not with BMF3 (Su et al., 2021), demonstrating that the interaction patterns between KNL1 and SAC proteins could be various in different plant species. Like in vertebrates, this region of KNL1 could form a helical conformation alongside a hydrophobic interface of the tetratricopeptide repeat (TPR) domain of BMF1/2 (Su et al., 2021).

#### 2.3.2 Chromosome passenger complex proteins

The loading and functioning of SAC in kinetochores depend on CPC, which comprises the core enzyme Aurora kinase and three non-enzymatic kinases, namely inner centromere protein (INCENP), Borealin, and Survivin (Komaki et al., 2020; Zhou et al., 2023). Plant Aurora kinases are classified into α-Aurora and β-Aurora, with higher plants possessing both types. For example, AURORA1 and AURORA2 in *Arabidopsis* and rice belong to α-Aurora, while AURORA3 belongs to β-Aurora (Demidov et al., 2005; Weimer et al., 2016). However, lower plants, such as moss and *Marsilea vestita*, contain only α-Aurora (Demidov et al., 2005). In plants, α-Aurora is found in spindle MTs during mitosis, while β-Aurora is found in kinetochores. Their characteristics and functions differ from those of Aurora A, Aurora B, and Aurora C in mammals (Komaki et al., 2020). Deng et al. reported that α-Aurora facilitated cytokinesis progression through phosphorylation-dependent restriction of microtubule-associated protein 65-3 (MAP65-3) associating with MTs in the phragmoplast midzone (Deng et al., 2024c). *Arabidopsis* CENH3 is a substrate of AURORA3, with serine 65 of CENH3 being phosphorylated preferentially in meristematic tissues, including flower buds and flowers, which is crucial for the proper development of reproductive tissues (Demidov et al., 2019).

INCENP is the largest non-catalytic subunit of CPC, directly binding to other CPC components in animals and yeast. Specifically, the N-terminal of INCENP interacts with Borealin and Survivin, while the C-terminal domain with four amino acid residues, known as the IN-box, binds to Aurora B (Komaki et al., 2020). Recently, a putative ortholog of INCENP, called *WYRD* (*WYR*), was found in *Arabidopsis* (Kirioukhova et al., 2011). WYR co-localizes with BORR (borealin-related, a homolog of Borealin) in the central region of kinetochores and the phragmoplast during mitosis and meiosis (Komaki et al., 2020). In addition, Komaki et al. identified *Arabidopsis* Survivin-like redundant proteins: borealin-related interactor 1 and 2 (BORI1 and BORI2) (Komaki et al., 2022). These proteins bind to phosphorylated histone H3, facilitating the correct association of CPC with the chromatin. The loss of BORI1 and BORI2 function is lethal, while their reduced expression causes severe developmental defects (Komaki et al., 2022).

### 2.4 Kinetochore proteins, cenDNAs and cenRNAs

Like most higher eukaryotes, plant cenDNA sequence is composed of functionally conserved but rapidly evolving tandem repeats (TRs) and centromeric retrotransposons (CRs) as outlined in **Table 2** (Oliveira and Torres, 2018; Han et al., 2021; Ramakrishnan Chandra et al., 2023). Centromeric TRs, which form satellite sequences through their multiple copies (Leo et al., 2020), constitute the main components of cenDNAs. These TRs are mostly 150-200 base pair (bp) long (about the length of a nucleosome DNA) and are rich in AT (Cutter and Hayes, 2015; Thakur et al., 2021). Chromatin immunoprecipitation followed by sequencing (ChIP-Seq) has been employed in numerous studies to identify CENH3-bound TR sequences, including *pAL1* (178 bp) in *Arabidopsis* (Wlodzimierz et al., 2023), *CentC* (156 bp) in maize (Gent et al., 2017), *CentO* (155 bp) in rice (Lv et al., 2024), *CL1* (177 bp) in radish (*Raphanus sativus*) (He et al., 2015), *HaCENH3CL124* (187 bp) in sunflower (*Helianthus annuus*) (Nagaki et al., 2015), etc. However, exceptions exist, such as the 20-bp-long *CentAs* bound by CENH3 in *Astragalus sinicus* (Tek et al., 2011), and the 5390-bp-long *St3-294* bound DNA in potato (*Solanum tuberosum*) (Gong et al., 2012). Interestingly, the length of TRs can vary even in the same species (Nagaki et al., 2011; Su et al., 2019). For example, tobacco (*Nicotiana tabacum*) contains TRs ranging from 48 to 92 bp (Nagaki et al., 2011).

**Table 2:** The cenDNAs and cenRNAs that bind to CENH3, CENP-C and KNL2 in plants.

Name	Species	DNA/RNA	Annotation	References
CENH3	*Arabidopsis thaliana*	*AthCEN178 (pAL1)*	178-bp tandem repeat	(Wlodzimierz et al., 2023)
	*Zea mays*	*CentC*	156-bp tandem repeat	(Gent et al., 2017)
	*Oryza sativa*	*CentO*	155-bp tandem repeat	(Lv et al., 2024)
	*Raphanus sativus*	*CL1*	177-bp tandem repeat	(He et al., 2015)
		*CL25*	348-bp tandem repeat	
	*Helianthus annuus*	*HaCENH3CL124*	187-bp tandem repeat	(Nagaki et al., 2015)
	*Astragalus sinicus*	*CentAs*	20-bp tandem repeat	(Tek et al., 2011)
	*Solanum tuberosum*	*St3-294*	5390-bp tandem repeat	(Gong et al., 2012)
	*Nicotiana tabacum*	*HT3E06*	92-bp tandem repeats	(Nagaki et al., 2011)
		*HT3G02*	68-bp tandem repeats	
		*HT1H04*	48-bp tandem repeats	
	*Saccharum officinarum*	*So1*	137-bp tandem repeat	(Huang et al., 2021)
	*Sorghum bicolor*	*SorSat137* (*CEN38*)	137-bp tandem repeat	(Kuo et al., 2021)
	*Glycine max*	*GmCent-1* family	92-bp tandem repeat	(Tek et al., 2010)
		*GmCent-4* family	411-bp tandem repeat	
	*Phaseolus vulgaris*	*CentPv1*	528-bp tandem repeat	(Iwata-Otsubo et al., 2013)
	*Vigna unguiculata*	*CentVu*	455-bp tandem repeat	(Iwata-Otsubo et al., 2016)
			721-bp tandem repeat	(Ishii et al., 2020)
			1600-bp tandem repeat	
	*Pisum sativum*	*TR-7*	164-bp tandem repeat	(Neumann et al., 2012)
		*FabTR-10-PST-A* family	459-bp tandem repeat	(Macas et al., 2023)
		*FabTR-10-PST-B* family	1975-bp tandem repeat	
	*Arabidopsis thaliana*	*ATHILA*	*Ty3* centromeric retrotransposon	(Wlodzimierz et al., 2023)
	*Zea mays*	*CRM*	*Ty3* centromeric retrotransposon	(Gent et al., 2017)
	*Oryza sativa*	*CRR*	*Ty3* centromeric retrotransposon	(Lv et al., 2024)
	*Saccharum officinarum*	*CRS*	*Ty3* centromeric retrotransposon	(Wang et al., 2022)
	*Glycine max*	*GmCR*	*Ty3* centromeric retrotransposon	(Tek et al., 2010)
	*Vigna unguiculata*	*VuCR*	*Ty3* centromeric retrotransposon	(Ishii et al., 2020)
	*Beta vulgaris*	*Beetle7*	*Ty1/copia* centromeric retrotransposon	(Kowar et al., 2016)
	*Brassica nigra*	*CL32*	*Ty1/copia* centromeric retrotransposon	(Wang et al., 2019)
KNL2	*Arabidopsis thaliana*	*pAL1*	178-bp tandem repeat	(Sandmann et al., 2017)
CENH3	*Zea mays*	*CentC* transcript	~900-nt	(Topp et al., 2004)
		*CRM* transcript		
CENP-C	*Zea mays*	*CentC* transcript	156-bp	(Du et al., 2010)
		small single stranded RNA	24-nt	
KNL2	*Arabidopsis thaliana*	*pAL1* transcript	178-bp	(Sandmann et al., 2017)
		small single stranded RNA	23-nt	

CRs are mobile elements mediated by RNA, which is retrotranscribed into DNA, and then transposed. They constitute a class of elements capable of moving within the genome, transcribing their RNA into DNA using reverse transcriptase, and subsequently inserting the DNA into a new genomic location. The insertion and relocation of these transposons in the genome facilitate the assembly of CENH3 nucleosomes, thus contributing to the formation of centromere and telomere (Presting, 2018). CRs, in higher plants, are mainly represented by *Ty3* and *Ty1/copia* retrotransposons, both belonging to the long terminal repeat (LTR) family. However, in most plants, CRs are predominantly *Ty3* retrotransposons. *Ty1/copia* have been reported in wheat (*Triticum aestivum*), *Brassica nigra*, *Nelumbo nucifera*, and the genus *Sorghum* (Li et al., 2013; Zhu et al., 2016; Wang et al., 2019; Kuo et al., 2021). Furthermore, *Ty3* in grasses, including maize *CRM*, rice *CRR*, and sugarcane (*Saccharum officinarum*) *CRS*, exhibit high homology (Gent et al., 2017; Wang et al., 2022; Lv et al., 2024).

The transcription of cenDNAs in plants was demonstrated by May et al. for the first time (May et al., 2005). Plant cenDNAs vary considerably between species and are often not conserved across different chromosomes within a single species. Hence, cenRNAs are not conserved as well. Centromeres are transcribed by RNA Polymerase II (Pol II) (**Figure 1**) (Grenfell et al., 2016), with kinetochore and spindle assembly being dependent on the low levels of cenRNAs (Corless et al., 2020; Leclerc and Kitagawa, 2021). These low levels of cenRNAs are regulated by transcription factors (TFs) to inhibit Pol II activity, which is achieved through RNAi and modifications of cenDNAs and cenRNAs (Gutbrod and Martienssen, 2020; Wong et al., 2020; Mellone and Fachinetti, 2021; Hoyt et al., 2022). Generally, cenRNAs could be processed into various forms of RNA, including small RNAs (sRNAs), long non-coding RNAs (lncRNAs), circular RNA (circRNAs), and DNA: RNA hybrids (Liu et al., 2021). For example, maize CENH3 could immunoprecipitate with *CentC* and *CRM* transcript ranging from 40 to 900-nt (Topp et al., 2004). Meanwhile, maize CENP-C interacts with DNA through 122 amino acids (aa) encoded by exon 9-12, which binds to *CentC* transcript and a small single-stranded 24-nucleotide (nt) RNA (ssRNA) homologous to *CentC* (**Figure 2C**, **Table 2**). This is consistent with a model suggesting that cenRNA enhances the stability of CENP-C by increasing its affinity for DNA near the CENH3 nucleosome binding site (Du et al., 2010). The C-terminal region of αKNL2 interacts with RNA and DNA *in vitro*, featuring potential DNA-binding domains flanking the CENPC-k motif that are crucial for centromere localization (Sandmann et al., 2017). Yalagapati et al. demonstrated that the flanking DNA-binding regions and the CENPC/CENPC-k motifs play important role in the centromere localization of plant CENP-C and αKNL2 (Yalagapati et al., 2024). Similarly, *Arabidopsis* αKNL2 binds to 23-nt ssRNA derived from the TR *pAL1* transcript, with the full-length *pAL1* transcript competing with *pAL1* DNA for binding to KNL2 (**Table 2**) (Sandmann et al., 2017). Additionally, nucleolar centromeric transcripts were found in maize (Koo et al., 2016), but without the observation of nucleolar CENP-C (Du et al., 2010), suggesting that the nucleolar assembly pathway remains unclear. Moreover, R-loops are three strand nucleic acid structures comprising an RNA: DNA hybrid and a displaced single-stranded DNA (Crossley et al., 2022). Centromeric circRNAs and R-loops are also involved in centromere function. For example, maize circRNAs derived from *CRM* were reported to bind to centromeres through R-loops (Liu et al., 2020, 2021). During kinetochore assembly, CCTT (CENP-C targeting transcript), a human lncRNA, could boost the recruitment of CENP-C to cenDNAs though R-loop and RNA-protein interaction (Zhang et al., 2022). However, centromeric lncRNAs in plants remain abstruse.

## 3 Applications of kinetochore proteins in plants

### 3.1 Induction of ploidy changes in plants

Kinetochore dysfunction leads to frequent ploidy changes in plants, encompassing the formation of haploid and polyploid (Kozgunova et al., 2019; Keçeli et al., 2020). Uniparental genome elimination often occurs in interspecies hybridization, resulting in the formation of uniparental haploid progeny (Ishii et al., 2016). This method is commonly used in actual crop production to speed up the process of crop breeding by obtaining double haploid plants (Ishii et al., 2016). Doubled haploid induction has been achieved using both *in vitro* (culture of immature male or female gametophytes) and *in vivo* (inter- and intra-specific hybridization, centromere-mediated haploidization) methods, while the systematic *in vitro* methods are species- and genotype-dependent and limited (Dwivedi et al., 2015; Mayakaduwa and Silva, 2023). On the contrary, the method by haploid-inducing line is relatively high-efficient and its effect is stable without genotype restriction. CENH3-mediated haploid induction is one of the *in vivo* techniques used for haploid doubling breeding (Dwivedi et al., 2015; Mayakaduwa and Silva, 2023). Through sequence substitution or point mutation of CENH3, it compensates for the mutation of *cenh3* to form a haploid-inducing line. Crossing this haploid line with wildtype (WT) plants removes the uniparental genome, resulting in a progeny that possess only the chromosomes of the wild-type parent thus inducing a haploid generation. In addition to the common advantages of the method based on haploid-inducing line, cytoplasm swapping by CENH3-mediated haploid induction facilitates efficient screening of unique nucleotype-plasmotype combinations, a rapid and precise method for assessment of the phenotypic effects of natural variation in organellar genomes (Flood et al., 2020). The utility of paternal haploids for the rapid swap of cytoplasmic male sterility (CMS) from the donor to recipient has been successfully demonstrated in maize (Bortiri et al., 2024) and broccoli (*Brassica oleracea*) (Han et al., 2024), and is elaborated in the commentary by Maruthachalam (Maruthachalam, 2024).

In recent years, advances in CENH3-mediated haploid induction methods have been made in *Arabidopsis* (**Table 3**) (Ravi and Chan, 2010; Karimi-Ashtiyani et al., 2015; Kuppu et al., 2020; Marimuthu et al., 2021), maize (Kelliher et al., 2016; Wang et al., 2021), wheat (Lv et al., 2020), cotton (*Gossypium hirsutum*) (Gao et al., 2020), switchgrass (*Panicum virgatum*) (Yoon et al., 2022) carrot (*Daucus carota*) (Meyer et al., 2023) and onion (*Allium cepa*) (Manape et al., 2024), but many explicit studies are focused on the model plant *Arabidopsis* [reviewed in (Thondehaalmath et al., 2021)]. For example, *GFP–tailswap* is a transgene in which the N-terminal of CENH3 is replaced with that of histone H3.3 encoded by AT1G13370 and fused with green fluorescent protein (GFP) (Ravi and Chan, 2010). Transferring *Arabidopsis GFP-tailswap* into *cenh3-1* embryo-lethal mutants restored wild type phenotypes of the plants (Ravi and Chan, 2010). These *GFP-tailswap* plants, producing only a small amount of pollen, were used as maternal parents and crossed with different ecotypes of *Arabidopsis*. About 25 to 45% of the surviving progeny produced haploids containing only wild-type chromosomes, with the rest being diploids and aneuploids (**Table 3**) (Ravi and Chan, 2010). The similar method was applied for haploid plants in maize (Kelliher et al., 2016). However, the *GFP-tailswap* method has been ineffective in crop plants, and outside of *Arabidopsis*, it has proven to be less effective, generally producing <1% haploids (Kalinowska et al., 2019). There are multiple other frequently-used approaches to manipulate CENH3 in plants: expressing CENH3 protein that contained amino acids substitution or small deletion in its conserved C-terminal HFD in a *cenh3*/*cenh3* null mutant (Kuppu et al., 2020; Meyer et al., 2023), using a heterozygous null mutation (*cenh3*/*CENH3*) constructed through CRISPR-Cas9 (Wang et al., 2021; Yoon et al., 2022), and producing plants with restored frameshift mutations or deletions in the endogenous copy of *CENH3* (Lv et al., 2020). Interestingly, Manape et al. proposed that unlike *Arabidopsis* (Ahmadli et al., 2023) and maize (Kelliher et al., 2016), a nominal reduction in *CENH3* expression using RNAi resulted in genome elimination when crossed with WT onion, providing a potential method for crop production where CRISPR/Cas9-based knockout generation is not feasible (Manape et al., 2024). A study of cotton CENH3 RNAi manipulation showed similar results, and the haploid-inducing effects of *in vitro* CENH3 inhibition were much the same to that of *in vivo* CENH3 RNAi technique (Gao et al., 2020). However, the centromere-based haploid induction technology requires genome editing, which is not realistic for crop breeding. Thus, the protein degradation strategy for haploid induction plant may be more feasible, such as nanobody-targeted ubiquitin proteasome-based degradation of EYFP-tagged CENH3 in *Arabidopsis* (Demidov et al., 2022). Notably, in maize, the integration of two *in vivo* haploid induction methods, namely CENH3-mediated haploid induction and the conventional breeding improvement based on the Stock6 germplasm, could rapidly and effectively improve the maternal haploid induction rates of maize inducer lines (Meng et al., 2022). Besides *CENH3*, *KNL2* mutants were also used as haploid inducers, where a point mutation in the CENPC-k motif of KNL2 was found sufficient to produce haploid-inducing lines (Ahmadli et al., 2023). Thus, conserved CENH3 and other kinetochore proteins provided potential targets for haploid induction in crop varieties. Moreover, heat stress treatments have been shown to increase the frequency of CENH3-mediated or *knl2* mutant-induced haploid generation (Ahmadli et al., 2023; Jin et al., 2023).

**Table 3:** CENH3-mediated haploid induction of *Arabidopsis thaliana* ^a^.

Mutant type	Cross (♀×♂)	Total plants analyzed	Haploids
*GFP–tailswap*	*GFP–tailswap* × WT Col-0	67	23 (34%)
	WT Col-0 × *GFP–tailswap*	116	5 (4%)
	*GFP–tailswap* × WT Ler	127	32 (25%)
	*GFP–tailswap* × WT Ws-0	22	10 (45%)
*GFP-CENH3*	*GFP-CENH3* × WT	164	8 (5%)
	WT × *GFP-CENH3*	112	0 (0%)
G83E	G83E × WT Ler	164	20 (12.2%)
E89K	E89K × WT Ler	34	15 (44.1%)
A136T	A136T × WT Ler	21	5 (23.8%)
T159I	T159I × WT Ler	97	19 (19.6%)
Δ6 line 7	Δ6 line 7 × WT Ler	70	18 (25.7%)
Δ6 line 2	Δ6 line 2 × WT Ler	23	2 (8.7%)
Δ33 line 8	Δ33 line 8 × WT Ler	25	4 (16.0%)
Δ33 line 18	Δ33 line 18 × WT Ler	83	14 (16.9%)
*GFP–tailswap*	*GFP–tailswap* (22°C) × WT Col-0 (22°C)	73	31 (42.5%)
	*GFP–tailswap* (30°C) × WT Col-0 (22°C)	55	53 (96.4%)
	*GFP–tailswap* (22°C) × WT Col-0 (30°C)	67	38 (56.7%)
G83E	G83E (22°C) × WT Col-0 (22°C)	116	6 (5.2%)
	G83E (30°C) × WT Col-0 (22°C)	48	30 (62.5%)
	G83E (22°C) × WT Col-0 (30°C)	68	9 (13.2%)

a *GFP–tailswap* replaces the N-terminal tail domain of CENH3 with the tail of conventional H3, using the H3.3 variant (encoded by AT1G13370) and is tagged with green fluorescent protein. *GFP-CENH3* represents transgenic green fluorescent protein-tagged CENH3.

G83E: GGA→GAG codon changed by TILLING; E89K: GAG→AAG; A136T: GCG→ACG; T159I: ACT→ATT.

Δ6 line 7, Δ6 line 2, Δ33 line 8 and Δ33 line 8 are constructed through CRISPR/Cas9.

Data used were from the references (Ravi and Chan, 2010; Kuppu et al., 2020; Ahmadli et al., 2023).

In addition to the production of haploids, the destruction of plant kinetochore proteins also led to the formation of polyploids. There are two different pathways to study polyploidy in nature: mitotic or somatic chromosome doubling and cytogenetics variation (Basit and Lim, 2024). Chemical such as colchicine and gaseous i.e. nitrous oxide have been deliberated as strong polyploidy causing agents (Basit and Lim, 2024). Although colchicine is the most widely used polyploidy-inducing agent, it is highly toxic to mammals and plants. Arshad et al. firstly reported paclitaxel and caffeine–taurine, the new colchicine alternatives for chromosomes doubling in maize haploid breeding with lower morphological and physiological by-effects (Arshad et al., 2023). At present, there are many methods to induce polyploid artificially, but the methods that can be used for large-scale production are limited, and there are still problems such as low frequency and high chimerism rate when inducing chromosome doubling. With the deeper kinetochore-related researches, it is proven that destruction of plant kinetochore proteins may also cause chromosome doubling, providing a new possible perspective for polyploidy. For example, the knockout of several kinetochore components in moss resulted in the failure of chromosome separation and cytoplasmic division, ultimately leading to the formation of polyploid cells (Kozgunova et al., 2019). In *Arabidopsis*, prolonged SAC activation triggers a cell cycle reset, leading to the formation of duplicate chromosomes, and the absence of nuclear division (Komaki and Schnittger, 2017). Nonetheless, studies on kinetochore-related polyploids are still scarce.

### 3.2 Synthesis of plant artificial chromosomes

Synthetic genomics, a novel paradigm to study chromosome characteristics and edit biological functions, offers a fertile platform in the realm of applied crop science (Schindler et al., 2018; Birchler and Swyers, 2020; Fachinetti et al., 2020). With the development of synthesis technology and a gradual reduction of cost, plant genome construction has garnered increasing interest from researchers (Puchta and Houben, 2023; Wang et al., 2024). For example, leveraging the specific binding of lactose repressor (LacI) to the lactose operator (LacO), artificial centromeres were successfully constructed by depositing the LacI-CENH3 fusion protein on *Arabidopsis* chromosomes harboring the LacO site, using CENH3, as the primary epigenetic marker (Teo et al., 2013). Besides, LexA, the highly conserved repressor protein, plays a significant role in regulating the stress response of cells when chromosomal DNA is severely damaged (Gao et al., 2023). In maize, the native CENH3 could be recruited to long arrays of LexA operator (LexO) repeats through LexA-CENH3 fusion protein, forming new heritable centromeres (Dawe et al., 2023). Notably, CENH3-mediated genome elimination can be used as a trigger for ring minichromosomes which could serve as an alternative tool for stacking multiple genes through gene targeting, similar to the use of PACs (Tan et al., 2023). However, the precise sequence organization and structure of centromeres in most plants remain obscure. Furthermore, there appears to be no correlation between plant kinetochore proteins and the natural centromere sequences introduced through genetic transformation (Phan et al., 2006). PACs are synthetic chromosomes tailored for various applications in plant biology and biotechnology, including genetic engineering, trait stacking, gene expression studies, chromosome stability research, crop improvement, and biopharmaceutical production. As plant kinetochore proteins are the key components of chromosomes, a thorough understanding of their composition, function, and regulation are crucial for the successful synthesis of PACs.

## 4 Conclusion and future perspectives

Kinetochore complexes in animals and yeast have been extensively studied for the past few years, with a few homologs being identified in plants. However, studies on plant kinetochore complexes are still in its infancy. Many kinetochore protein components have been identified in animals and yeast, but their homologs have not yet been identified in plants. Furthermore, the reported plant kinetochore proteins also require further exploration, particularly with regards to their interacting proteins and specific molecular regulation mechanisms. By exploring deeper plant kinetochores, researchers can uncover unique features and adaptations that contribute to the accurate transmission of genetic material during cell division in plants. Apart from CENH3, the interactions between/among other kinetochore proteins, cenDNAs and cenRNAs are rarely reported, and the current research on plant kinetochore complex is confined to a few plant species. The components of the kinetochore complex possess great potential and economic value. Research in this area has the potential to lead to technological breakthrough in the field of crop production and synthetic genomics.
